# Review of current incidents and risk calculations used in the Royal College of Australasian Pathologists Key Incident Management and Monitoring Systems - a system that could be used by all Australasian medical laboratories, and easily adapted to worldwide use

**DOI:** 10.11613/BM.2022.010702

**Published:** 2021-12-15

**Authors:** Stephanie Gay, Belinda Pope, Tony Badrick, Michael Whiley

**Affiliations:** 1 Key Incident Management and Monitoring System (KIMMS) program, Royal College of Pathologists of Australasia Quality Assurance Programs, Sydney, Australia; 2 Quality Department, Douglass Hanly Moir, Sydney, Australia; 3 Medical Services, NSW Health Pathology, Sydney, Australia

**Keywords:** extra-analytical phase, laboratory organisation and management, pre- and post-analytical incidents, risk matrix, quality assurance

## Abstract

**Introduction:**

The Royal College of Pathologists of Australasia Quality Assurance Programs (RCPAQAP) Key Incident Monitoring and Management Systems (KIMMS) program has found that some existing Quality Indicators are too broad or not well defined. The risk matrix in use does not allow changes in incident Detection or Probability. In 2020, a review was performed: what issues should KIMMS include as Key Incidents and how could risk measurement be improved?

**Materials and methods:**

Twenty-seven networked and stand-alone laboratories enrolled in KIMMS during 2020 were surveyed on 45 current and new indicators of risk in the total testing process. They were asked which indicators they considered were significant in causing patient harm. Existing risk matrices in use by members of the KIMMS Advisory Committee laboratories were reviewed regarding their size or structure (3x3 or 5x5) and the descriptions of consequences and probability.

**Results:**

Thirteen participants indicated 21 indicators should be monitored, and the KIMMS Advisory committee added a further 13 (11 from the remaining 24 and 2 new). Of the five risk matrices reviewed, all consistently used a 5x5 matrix to estimate Consequences *vs* Probability of harm. The KIMMS advisory committee added a third parameter to the calculation of Risk, Detectability.

**Conclusion:**

All 34 pre- and post- indicators should be monitored, covering all aspects of the total testing cycle other than analytical. The risk measurement can be improved by introducing a 5x5 risk matrix to evaluate harm (consequences x probability) and then evaluating risk by adding detectability; risk equals harm x detectability.

## Introduction

Since 2011 the Royal College of Pathologists of Australasia Quality Assurance Program (RCPAQAP) has been monitoring pre-and post-analytical incidents in the Quality assurance program (QAP) Key Incident Monitoring and Management Systems (KIMMS). The observed incidents are too broad in some cases and/or not well defined. The program includes allocating Risk to pre-and post-analytical incidents using the Failure Mode and Effects Analysis (FMEA) principles to assign a risk rating level to each of the identified incidents (*1*, *2*). Measuring Risk aims to encourage laboratories to identify root cause problems in pre-and post-analytical phases and act to reduce the Risk. These corrective actions should then be continuously monitored and modified to ensure the Risk remains at a clinically acceptable level. This approach recognises that not all errors have the same impact on patients or have the same Detectability. Errors that occur with high frequency may not significantly risk patient safety compared to some low-frequency errors.

This existing risk assessment used by KIMMS has set risk factors for each incident, calculated from the Consequences of the incident multiplied by the ease of Detection. The Consequences are the likely outcome to the patient of an incident and are immutable. Both values are assigned by the KIMMS Advisory Committee and do not consider any mitigating steps an organisation may have taken to reduce the Risk. Thus, in the current risk calculation, if an organisation puts more resources into detecting more incidents and thus more effectively manages these risks, the risk profile of the existing model increases rather than decreases. The new risk calculation should be such that when an organisation improves its processes, the Risk of patient harm is shown to decrease. Further to these aims was to have a process that could be adapted for use in other areas of a medical laboratory, not just in the KIMMS program.

A review of pre-analytical and post-analytical incidents for quality indicator monitoring and an assessment of methods used to calculate Risk were undertaken in 2020. There were two parts to this study – what issues should KIMMS include as Key Incidents and how could the way that Risk is measured be improved?

## Materials and methods

Twenty seven networked laboratories and stand-alone Australian laboratories participate in KIMMS. They were sent a list of 45 incidents using a link sent by email to a Microsoft Forms survey and asked to identify which incidents they thought should be monitored by KIMMS. They were specifically asked to identify those that were important to patient harm, not just those they could measure easily. Definitions needed to be concise.

The KIMMS calculation of Risk needed to be improved, so that it would better reflect changes to the Risk that occurred when laboratories undertook improvements to their systems and for it to be in a form that other health professionals and health administrators would easily understand. By comparing what each organisation already had in place with regards to the size of their risk matrix (3x3 or 5x5) and the definitions used for consequences and probability, and what the ISO 22367:2020 standard recommended, KIMMS intended to design a comparable framework (*3*). This should also make the process commutable to other areas of the medical laboratory both in Australasia and overseas.

## Results

Fifteen participants out of the twenty-seven surveyed (56%) responded. Of the 45 incidents presented, 21 were retained as more than 8 out of 15 responders (> 50%) believed they were important to monitor. The KIMMS committee members reviewed the remaining 24 and concluded that an additional 11 incidents were also important to monitor, as well as 2 new ones (incorrect transport/storage temperature/handling and error in transcription of patient demographics) (Table 1). The 13 indicators that were not included are shown in Table 2. They were rejected based on either not causing direct harm to the patient (*e.g.,* a fixable laboratory process incident) or because they were a duplicate of an included incident.

**Table 1:** Risk points and incidents to be measured by KIMMS program, and how they were included

**Risk Points**	**Specific Risk**	**Source of Risk being included**
Test request	Test request: Clarification of tests required	Committee
	Test request: Insufficient requester details or signature missing	Committee
Collection: Identification	Collection: Unlabelled specimen and/or request	Participants
	Collection: Insufficient patient ID specimen and/or request	Participants
	Collection: ID mismatch between specimen and/or request	Participants
	Collection: Specimen from wrong patient (WSIT)	Participants
Collection: Documentation	Collection: Essential collection date and/or time not provided or discrepant between specimen and request	Committee
	Collection: Essential signature missing or discrepant on transfusion sample and/or request	Participants
	Collection: Essential clinical indication for test not provided	Committee
	Collection: Essential specimen type/site not provided	Committee
Collection: Specimen	Collection: Incorrect patient preparation	Committee
	Collection: Incorrect specimen type or container or acid	Participants
	Collection: No specimen received	Participants
	Collection: Insufficient specimen	Participants
	Collection: Specimen incorrect fill leading to incorrect specimen:additive ratio	Participants
	Collection: Specimen clotted or other clotting issues	Participants
	Collection: Specimen contaminated	Participants
	Collection: Specimen haemolysed	Participants
	Collection: Specimen leaking	Committee
Collection: Transport, Storage and Handling	Transport and Storage: Incorrect transport/storage temperature/handling	Committee
	Transport and storage: Transport delay leading to specimen being too old to test	Participants
Test registration	Test registration: Incorrect unique specimen identifier	Committee
	Test registration: Patient ID wrong patient	Participants
	Test registration: Error in transcription of patient demographic information	Committee
	Test registration: Incorrect or missed tests	Participants
	Test registration: Incorrect or missing specimen type, site, collection time	Participants
	Test registration: Incorrect requesting or copy doctor	Participants
Analytical	Analytical: Internal laboratory process incident - unfixable	Participants
	Analytical: Within laboratory ID error	Committee
	Analytical: Intra or inter-laboratory specimen lost or misplaced - irreplaceable	Participants
	Analytical: Intra or inter-laboratory specimen lost or misplaced - replaceable	Participants
Post Analytical	Post Analytical: Failure of clinical handover high-risk (critical) results	Committee
	Post Analytical: Failure of clinical handover non-critical results	Committee
	Post Analytical: Amended report. Significant patient impact	Participants
KIMMS – Key Incident Monitoring and Management Systems.		

**Table 2:** Incidents not to be monitored by KIMMS

Test request: Test request not received
Test request: Test request cancelled
Test request: Test add-on not possible
Test request: Requestor or request form invalid
Test request: Patient ID incorrect, missing, illegible or incomplete
Test request: No collection performed patient not available or refused
Test request: Invalid request *i.e.,* test not available or inappropriate
Test request: Duplicate request or within minimum repeat limits
Specimen collection: Specimen damaged
Specimen collection: Sample contaminated - microbiology colonising organisms
Specimen collection: Extra sample received no tests requested
Lab registration: Patient ID incomplete
Analytical: Modified report non-critical supplemental information added
Analytical: Laboratory process incident – fixable
Analytical: Amended report - no patient impact *e.g.,* typo
KIMMS – Key Incident Monitoring and Management Systems.

The indicators are more specific than previously in use and are aligned to risk points in the request-test-report cycle (*4*). Not all organisations will be required to measure all the incidents, as the work environment affects the significance. For example, in community-based patient testing, reports sent to a wrong doctor can have a significant impact on patient care as notifications are only sent to those specified doctors, the referring and “copy to” doctors. In contrast, when patients are tested because of a hospital admission, reports can be accessed by all staff involved in patient care. Another example where there is differing significance depending on the context, is the availability of clinical notes. Detailed clinical notes can be more critical for ensuring the correct interpretation of results in genetics and molecular biology than clinical notes required for routine biochemistry.

Five different risk matrices were reviewed from New South Wales, Victoria, South Australia, Northern Territory and New Zealand. They were from both private and public organisations. They were all consistent, with five levels of Consequence and five levels of Probability. Four of the five had different definitions for Consequences that were aligned with various sectors within the organisation, and included Clinical, Financial, Work health and Safety *etc.*

On review of the definitions within the clinical laboratory, it was identified that even for a rare event, a large laboratory would expect to see them more often than a rare event in a hospital or surgery. It was important to consider the Detectability of an incident - the event may only be rare because it is not detected. Key Incident Monitoring and Management Systems has thus developed a 2 phase Risk analysis by multiplying Consequences by the Probability to calculate a Harm factor. This forms the basis of the Risk Matrix; see Table 3 (*4*).

**Table 3:** Risk matrix for calculation of Harm factor

**Consequence/Probability**	**Rare** **(< 1/year)**	**Unlikely** **(1 *per* year)**	**Occasional** **(1 *per* month)**	**Likely** **(1 *per* week)**	**Frequent** **(1 *per* day or more)**
Negligible (minimal) Delay, inconvenience	**1**	**2**	**3**	**4**	**5**
Marginal (minor) Recollection required	**2**	**4**	**6**	**8**	**10**
Significant (moderate) Delayed managment (non malignant) and/or medical treatment	**3**	**6**	**9**	**12**	**15**
Serious (major) Delayed diagnosis (malignant) and/or surgical treatment	**4**	**8**	**12**	**16**	**20**
Critical (catastrophic) Serious harm to multiple patients and/or patient death	**5**	**10**	**15**	**20**	**25**

Phase 2 involves an additional dimension that can be added to the traditional Harm score by considering the ability to detect a potentially adverse incident. Risk is equal to the Harm factor multiplied by the Detectability. Each component of the risk score is estimated on a gradient scale of 1-5, and the definitions are in line with current practice in Australasian laboratories. A summary of the three parameters is shown in Tables 4-6.

**Table 4:** Consequences scale

**Scale**	**Name**	**Definition**
1	Negligible/Minimal	Minimal, delay, inconvenience
2	Marginal/Minor	Recollect required
3	Significant/Moderate	Delayed management (non-malignant) and/or medical treatment
4	Serious/Major	Delayed diagnosis (malignant) and/or surgical treatment
5	Critical/Catastrophic	Serious harm to multiple patients and/or patient death

**Table 5:** Probability scale

**Scale**	**Name**	Example definition*
1	Rare	< 1/year
2	Unlikely	1 *per* year
3	Occasional	1 *per* month
4	Likely	1 *per* week
5	Frequent	1 *per* day or more
*Each laboratory would need to set their own definition depending on their size.		

**Table 6:** Detectability scale

**Scale**	**Name**	**Definition**
1	Detected	Almost all are detected
2	Most detected	/
3	Half detected	/
4	Most not detected	/
5	NOT detected	Almost none are detected

## Discussion

Key Incident Monitoring and Management Systems identified the need to introduce a Risk factor that reflected any improvements a laboratory makes and fitted with the current modes of measuring Risk, namely the use of a risk matrix. Improvements can be made either by reducing the Probability of the incident occurring and/or increasing their ability to detect an incident. This can be achieved using a 2 phase approach, a matrix to calculate a Harm factor (consequences x probability) and a further risk factor calculation (harm x detectability).

When applying the existing risk matrix to identified KIMMS pre-and post-analytical incidents, many of them are of moderate or high harm (*1*). By further using the scale of Detectability, the actual Risk can be evaluated. Laboratories will be able to lower their Risk Score by targeting high-risk areas. The current KIMMS model states that all labs have equal risk factors. In the new model, this is not the case. For example, a laboratory that uses the automatic assessment of haemoglobin, lipaemia and icterus (HIL index) would have a risk factor of ‘likely’ probability x ‘recollection’ harm x ‘detected’ detectability (4 x 2 x 1 = 8). In comparison, the laboratory that uses visual detection would have ‘likely’ probability x ‘recollection’ harm x ‘most not detected’ detectability (4 x 2 x 4 = 16). It is easy to make a case for introducing HIL index to the laboratory. An unlabelled request incident is another example. A laboratory that receives most of its requests as a hardcopy would have a greater frequency of unlabelled or missing requests. The Risk in the new system would be ‘occasional’ probability x ‘recollection’ harm x ‘detected’ Detectability (3 x 2 x 1 = 6), while a laboratory that has mainly electronic requests system will have a risk of ‘rare’ probability x ‘recollection’ harm x ‘detected’ detectability (1 x 2 x 1 = 2)

The concept of this risk factor, which considers both Probability and Detectability, can be applied to medical laboratories everywhere. Although this study only involved Australian KIMMS participants and Australia and New Zealand risk matrixes, the incidents covered by KIMMS are not unique to Australasia (*1*). Likewise, the initial risk matrix can be applied to analytical incidents within any medical laboratory. The consequence of an incorrect troponin result is far greater than the consequences of an incorrect chloride result. To reduce the harm factor, the troponin assay needs to be very robust.

## Conclusion

A total of 34 pre- and post- indicators should be monitored, covering all aspects of the total testing cycle other than analytical. The risk measurement can be improved by introducing a 5x5 risk matrix to evaluate harm (consequences x probability) and then evaluating risk by adding detectability; risk equals harm x detectability. Each organisation will assign for themselves the Probability of an event occurring and their ability to detect an adverse incident. The KIMMS program will specify the Consequences factor.

This new model allows laboratories to identify and monitor the Risk of errors and to put measures in place to lower this Risk rather than just focusing on the frequency of an incident in isolation. These improvements to identifying Risk will benefit the laboratory in providing a focus for quality improvement activities that will ultimately improve patient care and outcomes.
